# Amelioration of Mitochondrial Dysfunction-Induced Insulin Resistance in Differentiated 3T3-L1 Adipocytes via Inhibition of NF-κB Pathways

**DOI:** 10.3390/ijms151222227

**Published:** 2014-12-02

**Authors:** Mohamad Hafizi Abu Bakar, Mohamad Roji Sarmidi, Cheng Kian Kai, Hasniza Zaman Huri, Harisun Yaakob

**Affiliations:** 1 Department of Bioprocess Engineering, Faculty of Chemical Engineering, University Teknologi Malaysia, Skudai 81310, Malaysia; E-Mail: chengkiankai@cheme.utm.my; 2 Institute of Bioproduct Development, University Teknologi Malaysia, Skudai 81310, Malaysia; 3 Innovation Centre in Agritechnology for Advanced Bioprocessing (ICA), University Teknologi Malaysia, Skudai 81310, Malaysia; E-Mail: harisun@ibd.utm.my; 4 Department of Pharmacy, Faculty of Medicine, University of Malaya, Kuala Lumpur 50603, Malaysia; E-Mail: hasnizazh@ummc.edu.my; 5 Clinical Investigation Centre, 13th Floor Main Tower, University Malaya Medical Centre, Kuala Lumpur 59100, Malaysia

**Keywords:** adipocytes, mitochondrial dysfunction, inflammation, oxidative stress, insulin resistance, celastrol, nuclear factor kappa B (NF-κB)

## Abstract

A growing body of evidence suggests that activation of nuclear factor kappa B (NF-κB) signaling pathways is among the inflammatory mechanism involved in the development of insulin resistance and chronic low-grade inflammation in adipose tissues derived from obese animal and human subjects. Nevertheless, little is known about the roles of NF-κB pathways in regulating mitochondrial function of the adipose tissues. In the present study, we sought to investigate the direct effects of celastrol (potent NF-κB inhibitor) upon mitochondrial dysfunction-induced insulin resistance in 3T3-L1 adipocytes. Celastrol ameliorates mitochondrial dysfunction by altering mitochondrial fusion and fission in adipocytes. The levels of oxidative DNA damage, protein carbonylation and lipid peroxidation were down-regulated. Further, the morphology and quantification of intracellular lipid droplets revealed the decrease of intracellular lipid accumulation with reduced lipolysis. Moreover, massive production of the pro-inflammatory mediators tumor necrosis factor-α (TNF-α) and interleukin-1β (IL-1β) were markedly depleted. Insulin-stimulated glucose uptake activity was restored with the enhancement of insulin signaling pathways. This study signified that the treatments modulated towards knockdown of NF-κB transcription factor may counteract these metabolic insults exacerbated in our model of synergy between mitochondrial dysfunction and inflammation. These results demonstrate for the first time that NF-κB inhibition modulates mitochondrial dysfunction induced insulin resistance in 3T3-L1 adipocytes.

## 1. Introduction

In recent years, the roles of mitochondrial dysfunction-induced inflammation towards progression of insulin resistance, the forerunner of type 2 diabetes mellitus, have acquired important new dimensions [1,2,3,4]. Indeed, a number of studies have discovered that the impairments of mitochondrial functions in skeletal muscles, liver and adipose tissues of both human and animal disease subjects are etiologically associated with low-grade chronic inflammation [5,6]. In light of data indicating a pathophysiologic role of mitochondrial dysfunction in the occurrence of inflammation and insulin resistance, it is intriguing to hypothesize that the metabolic adaptations observed in these target tissues may affect the whole body metabolism. To a smaller extent, it is now becoming clear that the derangements of cellular inflammatory mediators are inextricably linked to the development of oxidative stress and reduced mitochondrial functions in insulin resistance state. Although the molecular details of such signaling remain enigmatic, several reports have suggested that mitochondrial stress leads to the intense oxidation of mitochondrial DNA, lipid and protein, resulting in the advancement of pro-inflammatory cytokines production in the tissues [1,5,7].

Adipose tissues are the group of heterogeneous mix of adipocytes, which constitute a wide range of fat tissues, immune cells, stroma-vascular cells, such as fibroblast, endothelial cells and pericytes [8]. The integrative roles of these tissues in controlling numerous metabolic signaling pathways are critical for maintaining energy homeostasis. The enlargement adipocytes mass and hyperplasia may lead to reduced oxygen supply with consequent hypoxia. These conditions result in an infiltration of macrophage, resulting in the enormous accumulation of various pro-inflammatory cytokines [9,10]. Increasing experimental and clinical evidences have clearly documented that adipose tissue is the most common sites of inflammation in the progression of obesity-related conditions [11,12]. The macrophage infiltration results in the immense production of reactive oxygen species (ROS) and oxidative stress, setting up a vicious cycle and disturbing the steady-state of body homeostatic control [7,13].

Augmented level of circulating cytokines, leukocyte infiltration and oxidative stress have been utilized as the most current model of obesity-linked adipose inflammation [11,14]. An elevation of non-esterified free fatty acid (FFA) level activate inflammatory signaling pathways of toll-like receptor (TLR) family and inflammasome through impairment of mitochondrial function in several peripheral tissues [2,15,16,17,18]. Overwhelmed production of ROS drives intense oxidation of lipid, proteins and mitochondrial DNA (mtDNA), compromising genes encoding respiratory chain complexes as well as disturbing the efficiency of oxidative phosphorylation as a whole [4,19,20]. In this regard, further experimental analyses should focus on the investigation of inflammatory pathways in modulating mitochondrial functions. The IκB kinase-β (IKK-β)/nuclear factor kappa B (NF-κB) pathway has been implicated in the early key event in the pathogenesis of insulin resistance [6,21]. Presumably through its ability to regulate a wide range of pathological process in insulin resistance, NF-κB dysregulation may directly contributes to variety of metabolic dysfunctions observed in peripheral tissues. Notably, high expressions of inflammatory gene in the context of mitochondrial dysfunctions are vital in enhancing the sensitivity of NF-κB binding site [22].

Celastrol is a quinone methide triterpenoid that derived from the root of *Tripterygium wilfordii* (Thunder of God Vine). This active compound possesses several biological activities, including antioxidant, anti-cancer, and anti-inflammatory [23,24]. The exact molecular mechanism for mode of action of this compound is poorly understood with largely unknown functions, but it was shown to act as a potent inhibitor of NF-κB transcription factors without affecting DNA-binding activity of activator protein 1 (AP-1) [25]. Recently, *in vivo* co-administration of celastrol in diabetic mice results in the improvement of insulin sensitivity, glycemic control and oxidative stress with significant enhancement of renal functions and structural changes [26]. Nevertheless, there is no current evidence reporting the exact role of celastrol on adipocytes with insulin resistance.

As mitochondrial dysfunction is strongly associated with the development of insulin resistance, we undertook a more focused approach at the cellular level to investigate the systematic roles of IKK-β/NF-κB pathways inhibition in alleviating mitochondrial dysfunction-induced insulin resistance in 3T3-L1 adipocytes. This study established an *in vitro* model of insulin resistance induced by oligomycin (ATP synthase inhibitor) on 3T3-L1 adipocytes. In this study, we observed the mechanistic effects of celastrol (NF-κB inhibitor) on the mitochondrial functions, lipolysis with intracellular lipid accumulation, glucose uptake activity, insulin-signal-transduction related molecules such as insulin receptor substrate 1 (IRS1), Akt/protein kinase B (PKB), Akt substrate of 160 kDa (AS160), glucose transporter 1 (GLUT1) and glucose transporter 4 (GLUT4). The levels of pro-inflammatory cytokines were also measured in the study, with an attempt to investigate that NF-κB pathway is the target for ameliorating mitochondrial dysfunction in the development of insulin resistance and type 2 diabetes.

## 2. Results and Discussion

### 2.1. Effect of Oligomycin and Celastrol on the Cell Viability and NF-κB Expression of 3T3-L1 Adipocytes

**Figure 1:**
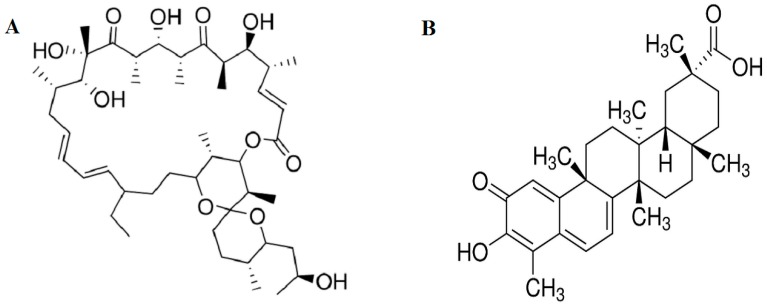

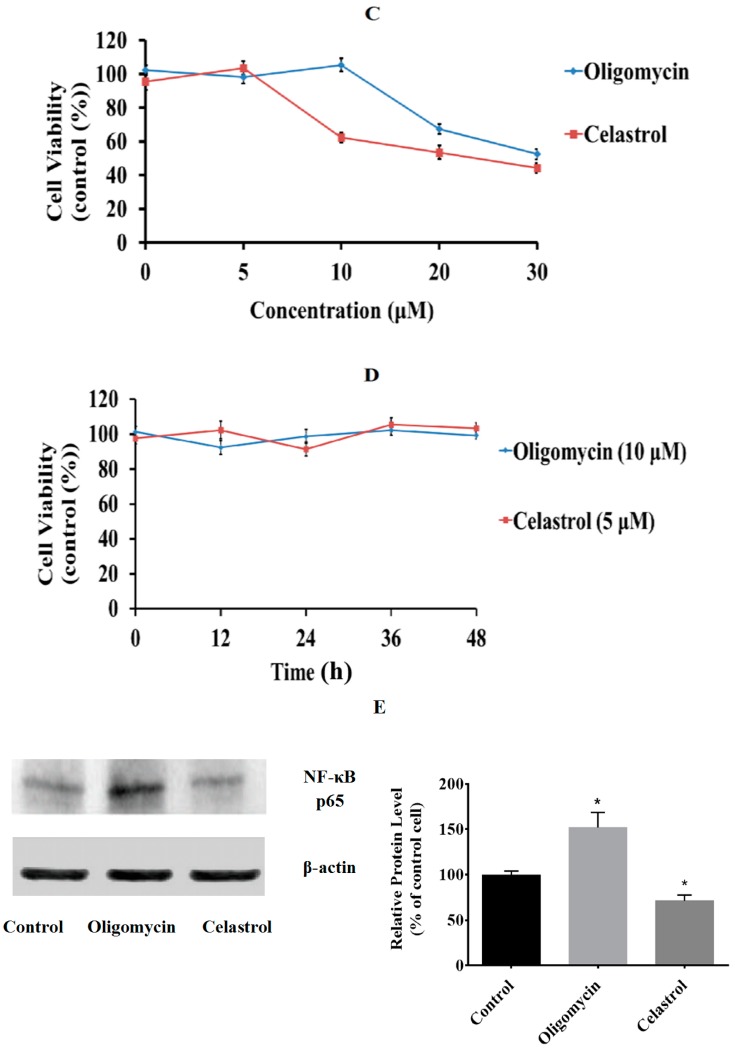
Effects of oligomycin and celastrol on cell viability. Chemical structures of (**A**) oligomycin and (**B**) celastrol were shown; The (**C**) dose-response and (**D**) time course of the cell viability for oligomycin and celastrol was determined via MTT assay. After serum starvation, the fully differentiated 3T3-L1 adipocytes were incubated with different doses of oligomycin (5, 10, 20 and 30 µg/mL) and celastrol (5, 10, 20 and 30 μM) in DMSO for 48 h. Time-course dependent (0, 12, 24, 36 and 48 h) was determined for each optimal dose. Effect of oligomycin and celastrol on nuclear factor kappa B (NF-κB) expression was performed via (**E**) immunoblotting analysis. β-actin was used as loading control. Protein levels measured were normalized against β-actin. Results were expressed as means ± SEM (*n* = 6–8). * *p* < 0.05 *vs*. untreated control.

We used differentiated 3T3-L1 adipocytes as an *in vitro* cellular model to study the ameliorative properties of celastrol on mitochondrial dysfunction induced by mitochondrial inhibitor oligomycin that can lead to insulin resistance. The viability assay was used to determine the suitable concentration and possible cytotoxic effects of oligomycin (Figure 1A) and celastrol (Figure 1B) treatments on fully differentiated 3T3-L1 adipocytes. After serum starvation, 3T3-L1 adipocytes were incubated in the presence of different doses of oligomycin and celastrol for 48 h. As shown in Figure 1C, both oligomycin and celastrol decreased the cell viability in a dose-dependent manner. The optimal dose of oligomycin that did not affect the cell viability was 10 μM. The cell viability of 3T3-L1 adipocytes incubated with celastrol discovered that the significant decline of cell viability was exhibited at the concentration of 10, 20 and 30 μM by 37%, 48% and 56%, respectively. Accordingly, optimal dose of 10 and 5 μM for oligomycin and celastrol were chosen in the assay, respectively. The time-course dependent was evaluated for each optimal dose (Figure 1D). The result depicted that 24 and 48 h treatment of both inhibitors on 3T3-L1 adipocytes did not significantly reduce the cell viability. Therefore, 48 h treatment of both inhibitor was chosen. Additionally, to confirm with our hypothesis on the involvement of NF-κB activation in the event of mitochondrial dysfunction, the protein expression of NF-κB level in both treated group was measured. The result showed that oligomycin significantly (*p* < 0.05) amplified NF-κB expression by 50% in the differentiated 3T3-L1 adipocytes whereas 29% reduction of NF-κB expression was exhibited by cells incubated with celastrol compared to control cells (Figure 1E).

### 2.2. NF-κB Inhibitor Recues 3T3-L1 Adipocytes from Oligomycin Induced Mitochondrial Dysfunction

**Figure 2:**
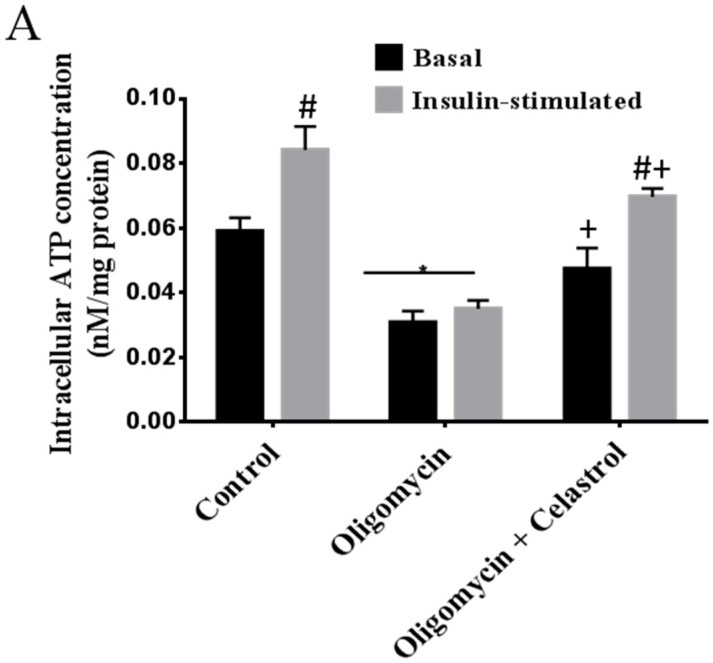

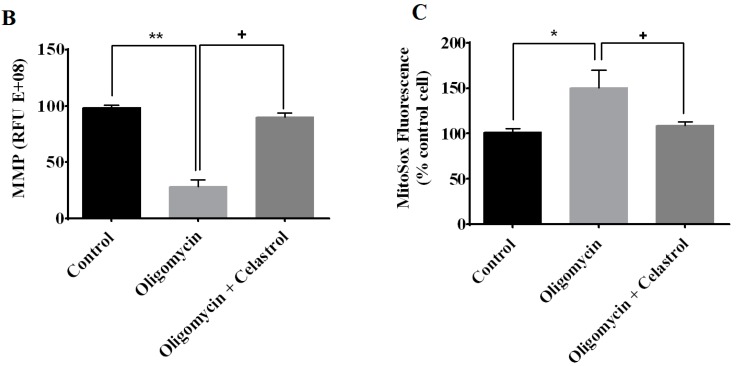
Effects of celastrol on mitochondrial function of differentiated 3T3-L1 adipocytes with mitochondrial dysfunction was assessed via (**A**) intracellular ATP concentration; (**B**) mitochondrial membrane potential (MMP) and (**C**) mitochondrial superoxide production. Basal level and insulin-stimulated condition are shown. Basal rate refers to the rate of glucose transport in the absence of insulin. Results are expressed as means ± SEM (*n* = 6). * *p* < 0.05 and ** *p* < 0.01 *vs*. untreated control; **^#^** *p* < 0.05 *vs*. basal rate; **^+^** *p* < 0.05 *vs.* oligomycin-treated cells.

As shown in Figure 2A, exposure of differentiated 3T3-L1 adipocytes to oligomycin for 48 h significantly decreased mitochondrial intracellular ATP concentration at the basal (48%, *p* < 0.05) and insulin-stimulated condition (58%, *p* < 0.01) compared to untreated control. After then, we inhibited the activation of NF-κB using 5 µM celastrol after exposure of 3T3-L1 adipocytes to oligomycin. The result revealed that celastrol significantly attenuated mitochondrial dysfunction in adipocytes treated with oligomycin by enhancing intracellular ATP concentration in the absence (28%, *p* < 0.05) and presence of insulin (41%, *p* < 0.05) (Figure 2A). To explore this relationship further, measurement of mitochondrial membrane potential was carried out. The results showed that oligomycin markedly (70%, *p* < 0.01) lessened the mitochondrial membrane potential of the adipocytes and this reduction was significantly inhibited by an escalation of 61% (*p* < 0.05) mitochondrial membrane potential after co-administration with celastrol for 48 h compared to control (Figure 2B). Mitochondrial superoxide production was determined with a fluorescence probe Mitosox™ Red (Invitrogen Molecular Probes, Carlsbad, CA, USA). Treatment of 3T3-L1 adipocytes with oligomycin significantly resulted in the elevation of ROS production by 50% (*p* < 0.05) compared to untreated control but was evidently diminished by celastrol treatment as much as 29% (*p* < 0.05) (Figure 2C).

### 2.3. Effect of Celastrol on Mitochondrial Dynamics of 3T3-L1 Adipocytes with Mitochondrial Dysfunction

**Figure 3:**
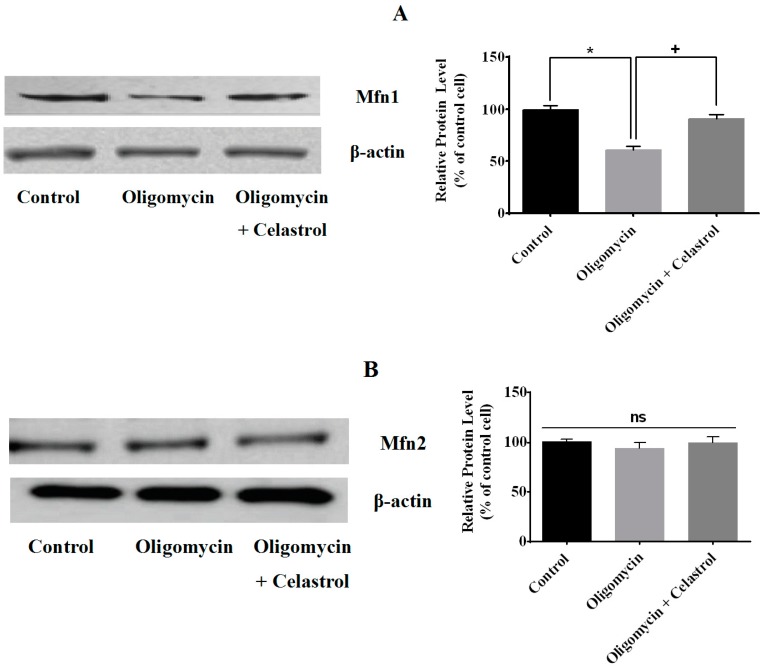

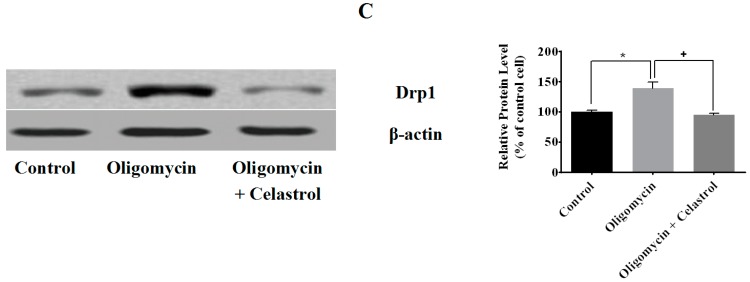
Effects of celastrol on mitochondrial dynamics of differentiated 3T3-L1 adipocytes treated with oligomycin. The mitochondrial fusion/fission protein levels in adipocytes treated with oligomycin, celastrol and control cells were analyzed by Western blot with the corresponding antibodies. Representative Western blot and densitometric analysis of Western blot results for (**A**) Mfn1, (**B**) Mfn2, and (**C**) Drp1 were determined. β-actin was used as loading control. Protein levels measured by densitometry were normalized against β-actin signals. The results are similar of those obtained from three independent experiments. * *p* < 0.05 *vs*. untreated control; **^+^** *p* < 0.05 *vs*. oligomycin-treated cells; ns: not significant.

Mitochondrial dynamics involving fusion and fission have been recognized as one of the key occasions in the mitochondrial metabolism. To study the involvement of NF-κB activation in adipocytes with mitochondrial dysfunction, we continued to examine the mitochondrial protein levels such as mitofusin 1 (Mfn1), mitofusin 2 (Mfn2), and dynamin-related protein 1 (Drp1), which are participated in mitochondrial dynamics process. Our data showed that oligomycin treatment lead to the significant declined of Mfn1 protein level by 40% in 3T3-L1 adipocyte, but was significantly re-amplified (30%, *p* < 0.05) by celastrol (Figure 3A). However, we found no significance difference on Mfn2 protein levels on adipocytes treated with oligomycin as well as co-treatment with celastrol (Figure 3B). Meanwhile, Drp1 protein level was augmented (38%, *p* < 0.05) in oligomycin treated cells compared to control cells and completely reversed by celastrol (Figure 3C).

### 2.4. Effect of Celastrol on Impairment of Mitochondrial Respiratory Chain Elicited Oxidative Stress in 3T3-L1 Adipocytes

**Figure 4:**
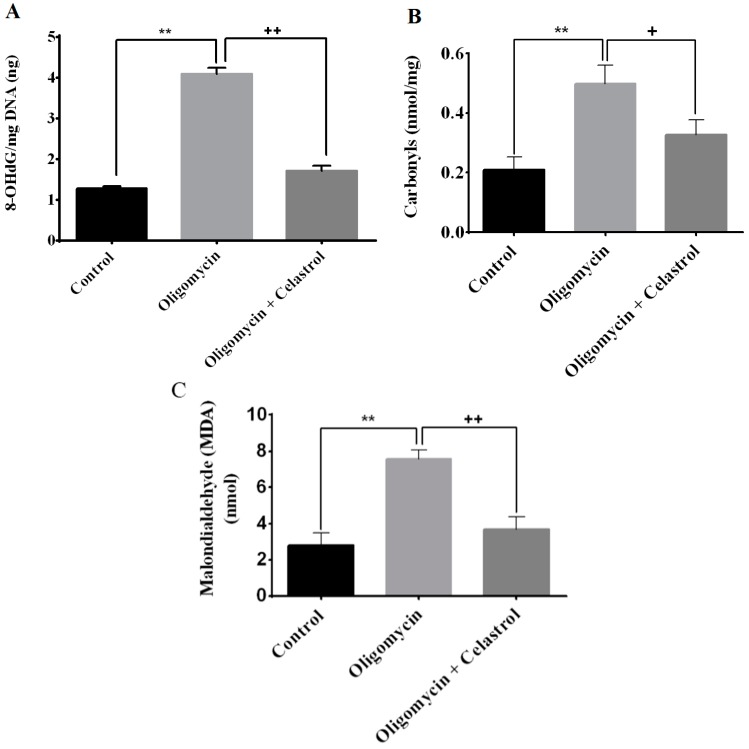
Effects of celastrol treatment on oligomycin-induced oxidative stress in 3T3-L1 adipocytes. Celastrol significantly reduced the oxidative stress of adipocytes with mitochondrial dysfunction. Oxidative profiles of adipocytes were determined via (**A**) measurement of DNA oxidative damage (8-OHdG); (**B**) protein carbonylation and (**C**) lipid peroxidation (MDA). Results are expressed as means ± SEM (*n* = 6). ** *p* < 0.01 *vs*. untreated control; **^+^** *p* < 0.05 and **^++^** *p* < 0.01 *vs*. oligomycin-treated cells.

Oxidative stress is a key link between inflammation and insulin resistance [6]. To address this association, the oxidative profile of adipocytes treated with oligomycin and its co-incubation with NF-κB inhibitor was evaluated. Oligomycin significantly multiplied the formation of 8-hydroxydeoxyguanosine (8-OHdG) by 3.21-fold (*p* < 0.01) in 3T3-L1 adipocytes. In parallel, our data evidenced that treatment with the celastrol on adipocytes with mitochondrial dysfunction significantly diminished the formation of 8-OHdG level by 1.87-fold (*p* < 0.01) (Figure 4A). The role of protein carbonylation in the pathogenesis of mitochondrial dysfunction and oxidative stress-related inflammation is well recognized [27]. Hence, calorimetric quantification of carbonylation of protein by-products was employed. In this study, we found that carbonylation of proteins was considerably (*p* < 0.01) elevated in oligomycin-treated cells than those of the control. The regulatory effect of celastrol on adipocyte with increased protein carbonylation was then further examined. Evaluation of protein carbonylation assay showed that co-incubation of oligomycin-treated adipocytes with celastrol reduced the protein carbonylation levels by 0.8-fold (*p* < 0.05) compared to oligomycin-treated cells (Figure 4B).

We then measured the lipid peroxidation of adipocytes with mitochondrial dysfunction induced by oligomycin treatment. The result revealed a significant upsurge of the lipid peroxidation (MDA) level in adipocytes treated with oligomycin compared to untreated control (270%, *p* < 0.01). In addition to the improvement of DNA oxidative damage and protein carbonylation, co-treatment with celastrol in oligomycin-treated cells exhibited with amelioration of lipid peroxidation level by 1.39-fold reduction (*p* < 0.01) (Figure 4C), signifying the enhancement of oxidative profile by inhibiting NF-κB activation may directly contribute to a significant role in rescuing adipocytes from the state of mitochondrial dysfunction induced by oligomycin.

### 2.5. NF-κB Inhibitor Reduces Lipolysis and Alleviates Accelerated Intracellular Lpid Accumulation in 3T3-L1 Adipocytes with Mitochondria Dysfunction

**Figure 5:**
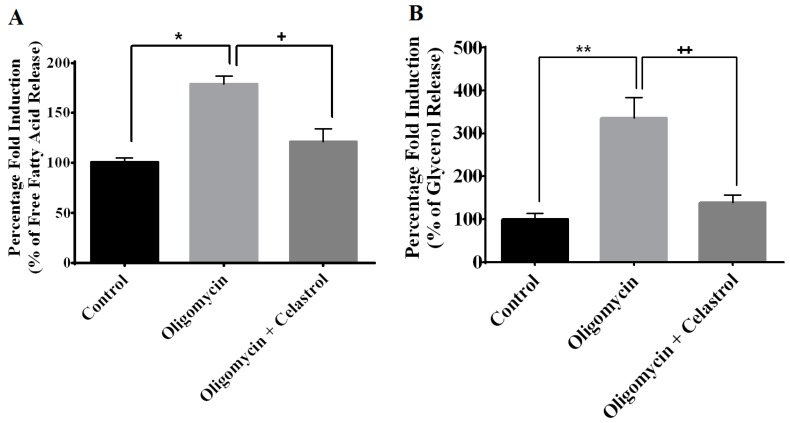

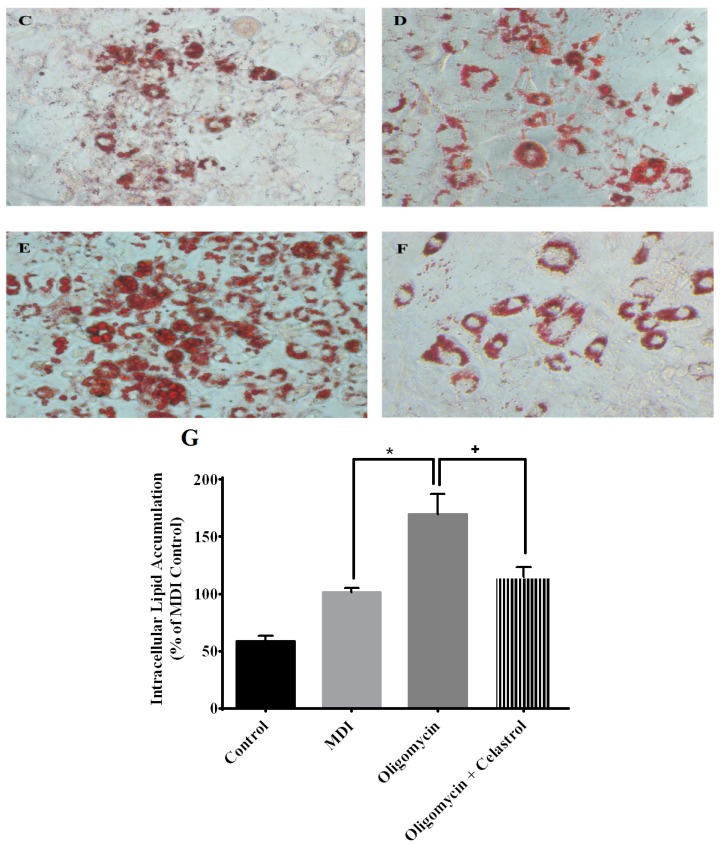
Effects of celastrol treatment on lipolysis and intracellular lipid accumulation of differentiated 3T3-L1 adipocytes with mitochondrial dysfunction. In the presence of MDI, fully differentiated 3T3-L1 adipocytes were incubated with oligomycin and celastrol for 48 h differentiated cells were on analyzed for (**A**) free fatty acids and (**B**) glycerol release as described in materials and methods. The Oil Red O stained adipocytes were photographed at 40× magnification; (**C**) Untreated control (DMSO); (**D**) MDI-treated cells; (**E**) Oligomycin-treated cells and (**F**) Oligomycin + celastrol-treated cells. Figures represent one of three independent experiments; and (**G**) Quantification of intracellular lipid droplets in 3T3-L1 adipocytes treated with oligomycin and celastrol was performed accordingly. The results are representative of those obtained from three independent experiments. All values are presented as means ± SEM of three independent experiments. * *p* < 0.05 and ** *p* < 0.01 *vs*. untreated cells; **^+^** *p* < 0.05 and **^++^** *p* < 0.01 *vs.* oligomycin-treated cells.

Lipolysis is the key metabolic process via engagement of the triglycerides breakdown into three molecules of free fatty acids and one molecule of glycerol. To determine the effect of NF-κB inhibitor on lipolysis in the context of mitochondrial dysfunction of adipocytes, the quantification of the free fatty acid and glycerol release was measured. Treatment with oligomycin on 3T3-L1 adipocytes significantly intensified the release of fatty acids and glycerol from adipocytes by 0.7- (*p* < 0.05) and 2.36-fold (*p* < 0.01), respectively, compared to untreated cells. NF-κB inhibition by celastrol leads to the pronounced reduction in free fatty acids (64%, *p* < 0.05) and glycerol (197%, *p* < 0.01) release compared to oligomycin-treated cells, signifying the down-regulation of lipolysis in the adipocytes with mitochondrial dysfunction could be modulated towards knockdown of NF-κB activation (Figure 5A,B).

Concomitant to lipolysis, we then focused on intracellular lipid accumulation to determine if celastrol has an effect on intracellular lipid deposition of 3T3-L1 adipocytes. The intracellular lipid accumulation in 3T3-L1 adipocytes treated with oligomycin and celastrol was assessed via Oil Red O assay (morphological and spectrophotometric quantification). It is worth noting that treatment of 3T3-L1 adipocytes with oligomycin significantly boosted the intracellular lipid accumulation. As visualized using Oil Red O staining, the formation of numerous cytosolic lipid droplets, larger and prominent increase in lipid vesicles with scattered and abnormal lipid droplet distributions were observed in 3T3-L1 adipocytes treated with oligomycin (Figure 5E) compared to the vehicle-treated cells with DMSO (Figure 5C) and MDI (3-isobutyl-1-methylxanthine, dexamethasone, and insulin) induction media (Figure 5D). The normal differentiated cells treated with MDI were exhibited with a typical adipocyte phenotype with round-shaped lipid droplets. In the quantitative examination using optical density at 490 nm, the addition of oligomycin in differentiated 3T3-L1 adipocytes was shown to up regulate intracellular lipid accumulation by 67% (*p* < 0.05) compared with MDI-treated cells (Figure 5G). Importantly, we demonstrated that the metabolic inhibition of NF-κB pathways by celastrol significantly (*p* < 0.05) reduced the intracellular accumulation of lipid droplets in adipocytes with insulin resistance by 52% as morphologically and quantitatively shown in Figure 5F,G.

### 2.6. NF-κB Inhibitor Suppress Production of Tumor Necrosis Factor-α (TNF-α) and Interleukin-1β (IL-1β) in the Event of Mitochondrial Dysfunction of 3T3-L1 Adipocytes

**Figure 6:**
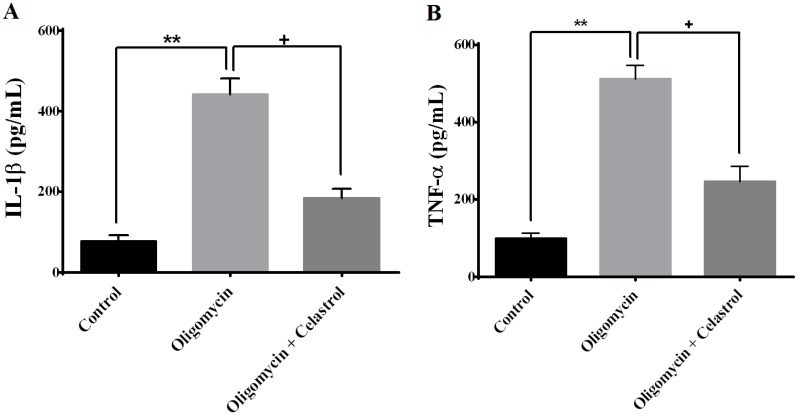
Effects of celastrol treatment on differentiated 3T3-L1 adipocytes treated with oligomycin. The measurement of pro-inflammatory cytokine (**A**) interleukin-1β (IL-1β) and (**B**) tumor necrosis factor-α (TNF-α) was analyzed using a commercially available ELISA kit. Results were expressed as means ± SEM (*n* = 8). ** *p* < 0.01 *vs*. untreated control; **^+^** *p* < 0.05 *vs*. oligomycin-treated cells.

IL-1β and TNF-α are major circulating cytokines that promote inflammatory action in adipocytes. As shown in Figure 6A,B, treatment with oligomycin on fully differentiated 3T3-L1 adipocytes for 48 h significantly led to the amplification of IL-1β (4.63-fold, *p* < 0.01) and TNF-α (4.15-fold, *p* < 0.01) secretion from adipocytes compared to untreated control. Celastrol significantly decreased the release of TNF-α (2.68-fold, *p* < 0.05) and IL-1β (3.37-fold, *p* < 0.05) from adipose tissue.

### 2.7. Co-Incubation of Celastrol Lead to the Restoration of Insulin-Induced Glucose Uptake Activity and Improved Insulin Signaling Cascades in Oligomycin-Treated 3T3-L1 Adipocytes

**Figure 7:**
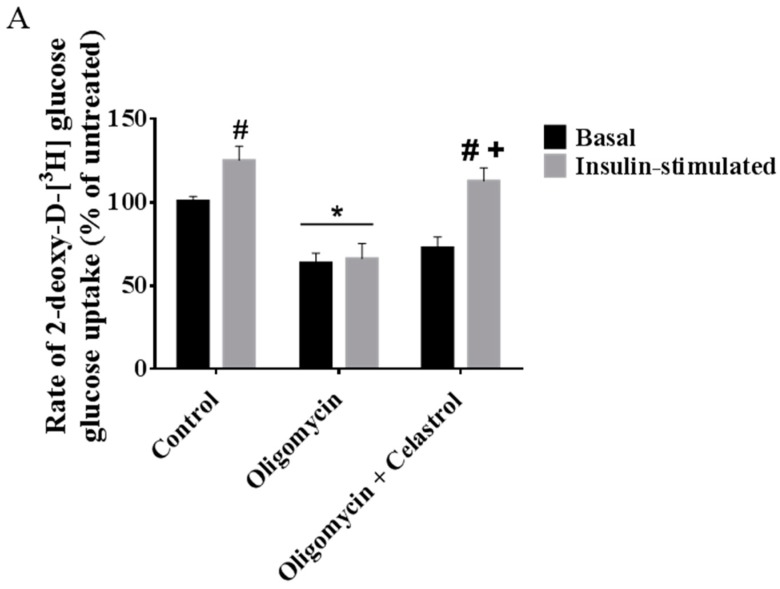

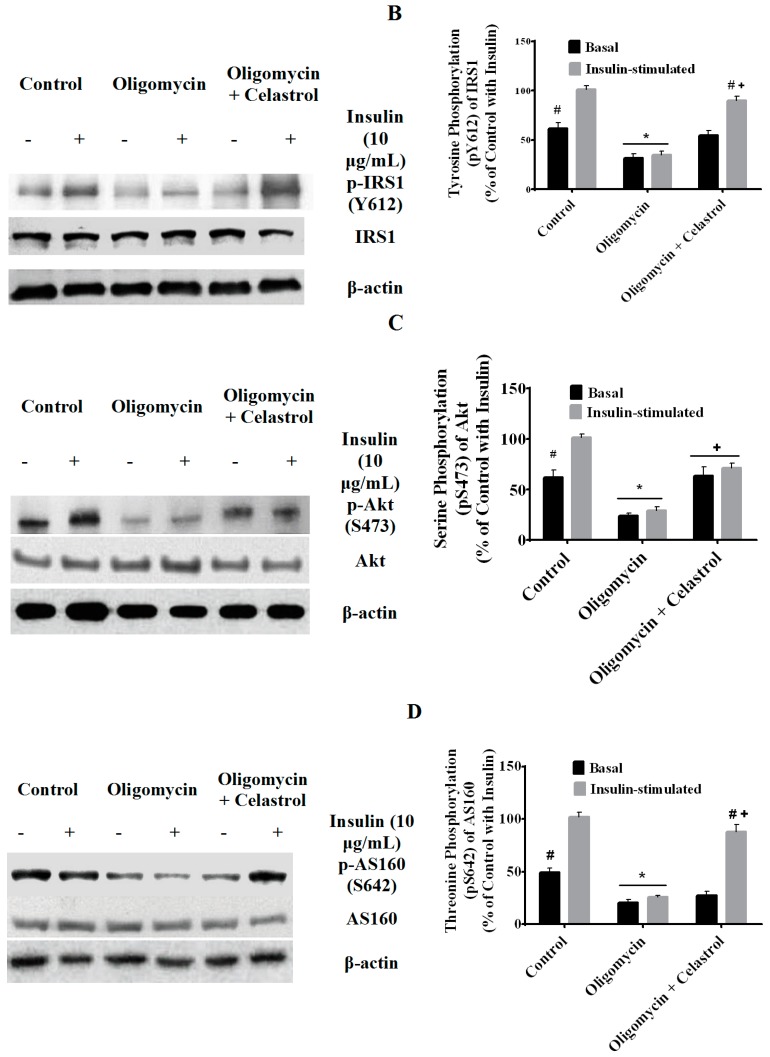

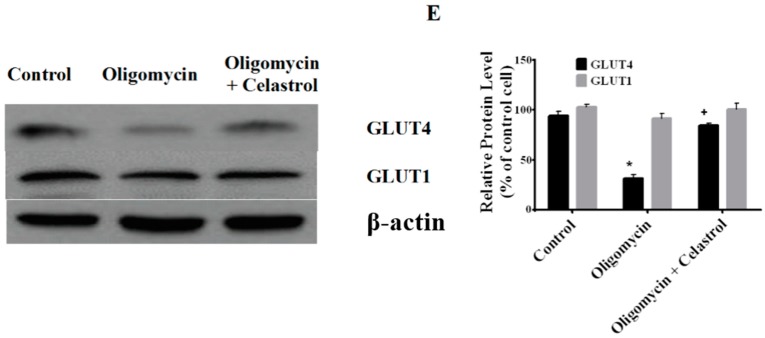
Effects of celastrol treatment on insulin signaling cascades and glucose transporter in differentiated 3T3-L1 adipocytes with mitochondrial dysfunction. Representative Western blot of insulin signaling activity for (**A**) glucose uptake; (**B**) Tyr612 phosphorylation of IRS1; (**C**) Ser473 phosphorylation of Akt; (**D**) Thr642 phosphorylation of AS160; (**E**) and expression level of glucose transporter (GLUT4 and GLUT1) in whole-cell lysates were determined. Fully differentiated 3T3-L1 adipocytes were incubated in serum-free DMEM overnight then treated with 10 µg/mL oligomycin and 5 µM celastrol for 48 h. Insulin (10 μg/mL) was added into plate for 30 min at 37 °C. β-actin was used as loading control. Protein levels measured were normalized against β-actin. * *p* < 0.05 *vs*. untreated control; **^#^** *p*< 0.05 *vs*. basal rate; **^+^** *p* < 0.05 *vs*. oligomycin-treated cells.

To investigate whether NF-κB activation may play a significant role in affecting glucose metabolism of adipocytes with mitochondrial dysfunction, we first looked at the event of glucose uptake activity of 3T3-L1 adipocytes with or without treatments. Cells in 24-well plates were treated for 48 h with oligomycin and celastrol in growth media in the presence or absence of insulin. The result showed insulin-induced glucose uptake was 0.24 times higher than that in normal basal group, but was reduced as much as 37% (*p* < 0.05) and 47% (*p* < 0.05) at the basal and insulin-stimulated condition, respectively, after incubation with oligomycin for 48 h. Co-treatment with celastrol partly reversed the glucose uptake activity of adipocytes with mitochondrial dysfunction. Insulin-induced glucose uptake was boosted by 37% (*p* < 0.05) after co-incubation with 5 μM celastrol for 48 h (Figure 7A), thus partly reverting the cell to normal phenotype relative to untreated control.

Given the importance of insulin signaling activity in adipose tissue, it is appealing to investigate the association of NF-κB pathways in the context of mitochondrial dysfunction by measuring insulin signaling activity and relative expression of glucose transporter protein in adipocytes. IRS1 is a protein effector that consists of multiple tyrosine phosphorylation motifs that mediate the metabolic growth of cell towards insulin stimulation. One of the important IRS1 tyrosine residues involved in the activation of phosphoinositide 3-kinase (PI3K) relative to insulin response is Tyr612. Akt, also known as protein kinase B is a serine/threonine protein kinase that regulates numerous signaling pathways including proliferation, survival and metabolism. Phosphorylation of serine 473 (Ser473) residues in the hydrophobic *C*-terminal regulatory domain is largely dependent on insulin stimulation. AS160 is a downstream effector of Akt activation in distal insulin signaling pathways that modulate the increased translocation of cell-surface GLUT4 in 3T3-L1 adipocytes. In the present study, we showed that the phosphorylation of IRS1, Akt and AS160 under insulin stimulation was completely lessened after incubated with oligomycin for 48 h. Meanwhile, co-treatment with celastrol under insulin stimulation significantly attenuated detrimental effect regulated by oligomycin through an elevated phosphorylation of these residue proteins (Figure 7B–D). Additionally, the expression of GLUT 4 was down-regulated while GLUT1 protein level was unaltered in adipocytes treated with oligomycin. Co-treatment of celastrol on adipocytes with mitochondrial dysfunction restored the level of GLUT 4 compared to control (Figure 7E).

### 2.8. Discussion

In recent years, emerging evidence has been gathered to support the notion that an increase of oxidative stress, mitochondrial damage and exacerbated inflammation are among the key features of obesity and type 2 diabetes [1,2,4]. The concerted actions of both acute and chronic inflammation with augmented superoxide free radicals productions can lead to further reduce the ATP generation, consequently impeding insulin-signaling activities in a number of peripheral tissues. Although it is broadly appreciated that oxidative stress and inflammation lead to development of insulin resistance, the therapeutic interventions in modulating these mitochondrial dysfunction-induced inflammations are relatively scarce. Therefore, further research in orchestrating other regulatory mechanisms and its ameliorative properties may provide an insight towards effective treatments of such disorders. Activation of redox-sensitive inflammatory pathways via NF-κB signaling by mitochondrial dysfunction has been postulated as an adaptive system of cellular stress towards overwhelmed generation of ROS [21]. One study has reported that the involvement of NF-κB pathways inhibits adipogenesis in murine cells [28] whereas the expression of adipocyte-specific genes were down-regulated by pre-treatment with TNF-α via NF-κB pathways [29]. Nevertheless, the precise mechanism linking NF-κB activation and mitochondrial dysfunction in adipocytes are still rather ambiguous.

Following the recent correlation between mitochondrial dysfunction and inflammation, the present study envisaged to further determine the mechanistic effect of a major regulator inflammatory response drug, celastrol upon treatment of common mitochondrial respiratory enzyme inhibitors oligomycin on fully differentiated 3T3-L1 adipocytes. We established an *in vitro* model of mitochondrial dysfunction in adipocytes through impairment of ATP synthase by 10 μM oligomycin treatment for 48 h. In parallel, to discover the involvement of NF-κB signaling pathways activation, we co-incubated oligomycin-treated cells with 5 μM celastrol for 48 h. The dose response and time course of the cell viability were performed accordingly in order to avoid possible toxic effects of oligomycin and celastrol (Figure 1C,D). After treatment with oligomycin, we confirmed earlier findings that mitochondrial dysfunction directly affects oxidative metabolism of adipocytes in the absence and presence of insulin [30,31]. Furthermore, with the use of this *in vitro* model, we observed a marked improvement of metabolic functions of 3T3-L1 adipocytes with mitochondrial dysfunction after treatment with celastrol. Our data in this study are the first to implicate the role of NF-κB inhibition in a mechanism of insulin resistance in adipocytes induced by mitochondrial dysfunction.

Studies have recently demonstrated an association of mitochondrial dysfunction in adipocytes of subjects with a positive family history of diabetes, and in animals with obesity-associated type 2 diabetes [32,33]. Accordingly, uncontrolled generation of ROS in the event of mitochondrial dysfunction in other cell types have been linked to the stimulation of inflammatory signaling pathways via NF-κB activation [22]. Low-grade inflammatory and matrix degradation responses via mitochondrial Ca^2+^ exchange, ROS generation, and NF-κB activation may also contribute to the development of mitochondrial dysfunction [20]. The induction of mitochondrial dysfunction via inhibition of mitochondrial ATP synthase, oligomycin, significantly potentiates the cytokine-induced inflammatory response in normal synovial fibroblasts with an increase of PGE_2_ and IL-8 release. The authors evidenced that this inflammatory mechanism is regulated via ROS production and NF-κB activation [34]. In other cell types, the impairment of mitochondrial functions was also resulted in over-production of ROS, leading to the cytotoxicity progression with an accumulated inflammatory cytokines [35]. Consistent with these findings, a recent study utilizing integrative approach combining *in vivo* and *in vitro* revealed that an augmented expression of inflammatory genes is implicated in the enhanced sensitivity of NF-κB binding site to the promoter region of the cell in the context of mitochondrial dysfunction [22,36]. Similarly, with regard to the latter possibility in the mechanism of mitochondrial dysfunction, we have determined the effects of celastrol on differentiated 3T3-L1 adipocytes treated with oligomycin. A significant enrichment of intracellular ATP concentration with reduced ROS production was observed. In addition, an elevated mitochondrial membrane potential was detected in the celastrol-treated cells with oligomycin, suggesting the involvement of NF-κB signaling pathways in modulating such metabolic perturbations (Figure 2A–C).

As reduced mitochondrial functions with altered morphology are associated with mitochondrial dynamics, we next investigated the effects of celastrol on the expression of proteins responsible for mitochondrial fusion and fission. Mitochondria are dynamic organelles that frequently undergo fusion and fission in order to exchange power and content. The aberrant mitochondrial function indicates an imbalance of the cellular bioenergetics affecting mitochondrial fusion and fission. Mfn1 and Mfn2 are the key enzymes that involve in mitochondrial fusion via GTPase activity [37] while Drp1 is the main target protein participates in the mitochondrial fission in regards to mitochondrial division, stress response and apoptosis [38]. At present, the protein levels of Mfn1 and Drp1 were up-regulated in the events of mitochondrial dysfunctions of adipocytes while mfn2 protein level remained unchanged after treatment with oligomycin (Figure 3A–C). Gao and colleagues observed that the metabolic expression of Mfn1 and Drp1 in 3T3-L1 adipocytes was greatly altered in the high glucose and high free fatty acids while the level of Mfn2 was unaltered after all treatments [16]. Later, the same group also identified treatment with TNF-α on 3T3-L1 adipocytes affects mitochondrial dynamics of Mfn2 and Drp1 level whereas the expression of Mfn1 protein level was not affected [15]. The differences of the findings in both studies were not fully elucidated, as the metabolic regulation of mitochondrial fusion and fission are too complex to be understood. The authors believed that the changes in mitochondrial fusion and fission here are still worthy of further detailed investigation. Recently, another report [39] suggested that enforced mitochondrial hyperfusion by expressing a dominant-negative mutant of Drp1 or of MARCH5 resulted in the activation of NF-κB inflammatory signaling pathways in an activated kinase-1 (TAK1) and IKK-dependent manner, through the mitochondrial E3 ubiquitin ligase activator of NF-κB (MULAN). The study has concluded that mitochondria through mitochondrial fusion and fission convert the stress signal into cellular response via activation of NF-κB pathways [39]. This is consistent with our result on identifying the roles of NF-κB activation in mitochondrial dynamics of adipocytes with mitochondrial dysfunction. We observed that co-incubation of celastrol in oligomycin-treated cells significantly increased and reduced the expression of Mfn1 and Drp1 protein level, respectively, while expression of mfn2 was unaffected (Figure 3A–C).

It is of paramount importance to understand that emerging studies have started to propose the inflammatory signaling pathways mediated by activation of IKK-β/NF-κB pathways and other transcription factors are the central arbitrator in understanding oxidative stress responses in obesity-related conditions [6]. In view of that, a report utilizing obese *db*/*db* mice found that enhanced NF-κB activity was coupled with intensified oxidative stress as demonstrated by abnormal accumulation of mitochondrial superoxide productions [40]. Likewise, this complex interaction is also evidenced by our data showing that NF-κB inhibitor significantly improved the oxidative metabolism of adipocytes treated with ATP synthase inhibitor through a reduced oxidative stress in adipocytes with mitochondrial dysfunction. The accumulation of cellular DNA damage either from nucleus and mitochondria is thought to contribute to aging process mediated via changes in redox status and oxidative stress-induced inflammatory responses [41]. Several studies have revealed that diminished mitochondrial oxidative phosphorylation and ATP production following mtDNA damage, and oxidative stress are collectively contribute to the co-regulation of NF-κB and inflammasome-mediated inflammatory responses activation, precede the development of type-2 diabetes mellitus [42,43,44]. The involvements of advanced glycation end (AGE) products may also affect the redox status of inflammatory signaling pathways. Treatment with glycolaldehyde-derived AGE3 on human aortic endothelial cells results in the elevated 8-OHdG formation and TNF-α concentration. The authors also observed that an inhibition of NF-κB activation suppressed AGE3-induced 8-OHdG formation, implying that sustained activation of NF-κB might be play pivotal roles for endothelial dysfunction in diabetes [45].

Carbonylation is a type of protein oxidation involving non-reversible direct oxidation of the side chains of cysteine, histidine, and lysine residues by lipid peroxidation end products such as 4-hydroxy and 4-oxononenal [46]. An elevation of protein carbonylation is observed in the subcutaneous human adipose tissue [47] and adipose tissue of diet-induced obese mice [48]. The upsurge of protein carbonylation level was revealed to play a major instigating role in the inflammation related pathways. Chronic treatment of 3T3-L1 adipocytes with pro-inflammatory cytokines TNF-α lead to development of mitochondrial dysfunction, protein carbonylation and lipid peroxidation [48]. This is supported by another observation showing the oxidation of thioredoxin (a class of small redox proteins) that result in the release and activation of apoptosis signal-regulating kinase 1 (ASK1) have been linked to progression of mitochondrial dysfunction-induced inflammation. This process initiate a cascade of phosphorylation events culminating in the activation of c-Jun *N*-terminal kinases (c-JNKs) and the NF-κB pathway as well as serine phosphorylation of IRS1, giving rise to the formation of oxidized lipid and proteins [49].

Lipid peroxidation is an activated metabolic process that signals the redox imbalance causes the activation of intracellular inflammatory signaling pathways. 4-hydroxy-trans-2-nonenal (HNE), malondialdehyde and acrolein are among the common by-products of lipid peroxidation that were shown to activate various signaling intermediates and regulate cellular dysfunctions involving redox-sensitive transcription factor NF-κB [50]. A study utilizing Male Sprague-Dawley rats has shown that inhibition of lipid peroxidation by administration of raxofelast results in a significant reduction of MDA and declined myeloperoxidase (MPO) activity through suppression of pancreatic NF-κB activation and the TNF-α mRNA levels [51]. Moreover, another study signified that the activation of a redox-sensitive transcription factor NF-κB signaling pathways in the event of lipid peroxidation lead to the posttranslational modification of proteins and DNA leading to cytotoxicity and genotoxicity [50]. These observations were in parallel with our data showing that amplified level of DNA oxidative damage, protein carbonylation and lipid peroxidation was observed in the oligomycin-treated cell but inhibited by celastrol treatment for 48 h (Figure 4A). These evidences indicated that celastrol is a potent inhibitor of oxidative stress-induced activation of NF-κB in 3T3-L1 adipocytes.

Inadequate suppression of lipolysis in adipose tissues and boosted delivery of free fatty acids into ectopic tissues (e.g., muscle, pancreas and liver) play a key role in the exacerbation of insulin resistance [2]. Several pro-inflammatory cytokines has been shown to induce enhanced lipolysis in adipocytes. Co-treatment of 3T3-L1 adipocytes with IL-6 lead to augmented lipolysis with diminished ATP content [52]. The stimulatory effects of TNF-α on the cellular lipolysis was also documented via the mitogen-activated protein kinase (MAPK) family involving extracellular signal-related kinase (ERK) and JNK in different type of cells [53]. Incubation of primary cultures of human adipocytes in the presence of a cell-permeable peptide that inhibits NF-κB signaling showed the abolishment of the nuclear translocation of NF-κB in the event of TNF-α-induced lipolysis [54]. Our data contributes to a new finding that an intensification of lipolysis through the impairment of adipocyte ATP synthase may be regulated by the activation of NF-κB signaling pathways (Figure 5A,B).

Furthermore, the data presented in this study also demonstrated that suppression of IKK-β/NF-κB signaling pathway by celastrol may also give rise to the diminished intracellular accumulation of lipid droplets in 3T3-L1 adipocytes treated with oligomycin (Figure 5C–G). Abnormal lipid deposition in various peripheral tissues has been proposed as one of detrimental occasions in the progression of obesity and insulin resistance-related diseases [3]. In normal physiological conditions, the relative levels of fatty acids are constantly maintained at the balance of utilization and deposition. Abnormal accumulation of lipids due to enlarged adipocytes mass resulted in a disproportionate rate of fatty acid oxidation, causing the cellular deposition of various lengths of acylcarnitine fatty acids. The incomplete fatty acid oxidation in mitochondria is prominent to be observed as the efficiency of mitochondrial functions was blunted. It is not surprising that treatment of adipocytes with oligomycin may result in excess intracellular lipid accumulations due to aberrant mitochondrial functions to metabolize these lipid intermediates. The dysfunctional lipid storage in adipocytes are significantly correlated to the downstream effects of TLR4 stimulation via activation of NF-κB transcription factor, which then triggers the transcription of many pro-inflammatory genes [55].

Adipose tissue is now appreciated as one of the important endocrine tissues that secrete hormones and circulating cytokines such as TNF-α and IL-1β. The relative level of these pro-inflammatory cytokines were elevated in obese and diabetic people [14]. High levels of recombinant TNF-α and IL-1β in co-cultured 3T3-L1 adipocytes with primary peritoneal mouse macrophages were identified to inhibit the expression of GLUT4 in 3T3-L1 adipocytes [56]. Notably, excessive production of pro-inflammatory cytokines is characterized by defective mitochondrial functions, oxidative stress with over-generation of ROS [7]. Moreover, the activation of transcription factor NF-κB was proved to be a target of oxidative stress. Direct exposure of H_2_O_2_ in certain cell types was shown to be an effective inducer of NF-κB activation [57]. The linkage of oxidative stress and NF-κB activation are connected to the elevated production of pro-inflammatory cytokines that lead to inhibition of intracellular insulin signaling activities in a number of peripheral tissues [5]. These data suggest that elevated pro-inflammatory cytokines are strongly connected to the onset of oxidative stress induced insulin resistance. Our study showed that the concentration of IL-1β and TNF-α was higher in adipocytes with mitochondrial dysfunction than untreated cells. The pharmacologic modulation of NF-κB activation with celastrol significantly prevented up-regulation of TNF-α and IL-1β level in oligomycin-treated cells, supporting the previous findings that NF-κB regulate the enhanced expression of IL-1β and TNF-α correlated with mitochondrial dysfunction [1]. These results have at least provided solid evidence to substantiate that inhibition of IKK-β/NF-κB inflammatory signaling pathways play a role in modulating mitochondrial dysfunction-induced insulin resistance in 3T3-L1 adipocytes with inflammation (Figure 6A,B).

On the other hand, the attenuation effects of adipocytes with mitochondrial dysfunction by celastrol in this study are not limited to the abovementioned findings, but also significantly implicated in the restoration of insulin induced-glucose uptake and enhanced insulin-signaling activity (Figure 7A–D). It has been established that adipose tissues with mitochondrial dysfunction that occurs in the metabolic syndrome are manifested with impaired glucose homeostasis and insulin insensitivity [32,34]. The progressive enlargement of adipocytes mass with an infiltration of macrophage may lead to the over-production of pro-inflammatory cytokines with hypoxia-induced a decline in glucose utilization rate [58]. Multiple *in vitro* studies revealed that impaired glucose homeostasis relatively results from reduced mitochondrial functions. Co-treatment of 3T3-L1 adipocytes with high concentration of glucose and free fatty acids was shown to stimulate mitochondrial dysfunction [16]. In addition, a significant decrease in insulin-stimulated glucose uptake was observed in 3T3-L1 adipocytes with mitochondrial dysfunctions after four days exposure of pro-inflammatory cytokine TNF-α [15]. Another group showed that [31] the induction of mitochondrial dysfunction and over-production of ROS in adipocytes by adding mitochondrial inhibitors and knockdown of mitochondrial transcription factor A (mtTFA), respectively result in the lowered glucose uptake and impaired insulin signaling activity upon insulin stimulation in adipocytes. They also noticed that the expression of GLUT4 and secretion of adiponectin was significantly blunted in adipocytes with mitochondrial dysfunctions. Mauro and colleagues have proposed that an activation of NF-κB may affects the cellular reprogramming of bioenergetics pathways involving mitochondrial respiration and glucose utilization [59]. Our results first illustrated that inhibition of NF-κB pathways by incubating celastrol in 3T3-L1 adipocytes with mitochondrial dysfunction improved glucose uptake activity. Enhanced insulin signaling pathways were significantly observed via boosted phosphorylation of Tyr612 residue of IRS1, Ser473 residue of Akt/PKB, Thr642 residue of AS160 (Figure 7A–D), possibly implying this inflammatory signaling pathway is implicated in the pathogenesis of insulin resistance in adipose tissue with mitochondrial dysfunction.

The relative translocation of glucose transporter over the plasma membrane from intracellular storage vesicles within insulin-sensitive tissues is a major regulatory process for the maintenance of blood glucose homeostasis. GLUT4, also called the “insulin-regulated glucose transporter”, is one of the key proteins involved in the glucose uptake activity relative to insulin stimulation through an ATP-independent, facilitative diffusion mechanism. High expression of this membrane protein was evidenced in adipose tissues and skeletal muscle [60]. Particularly, reduced expression level of GLUT4 is associated with the pathogenesis of type 2 diabetes where transgenic mice with severe alteration of GLUT4 level developed insulin resistance with impaired glucose homeostasis [61]. Our result revealed that an inhibition of ATP synthase in adipocytes was led to a significant depletion of GLUT4 level (Figure 7E). Concurrent increase of ROS production has been suggested as one of the metabolic insults that down-regulate the expression of GLUT4 level upon oligomycin treatment [31]. Oxidative stress elicited by mitochondrial dysfunction may contribute to the insulin insensitivity of adipocytes. Co-incubation with celastrol on 3T3-L1 adipocytes with mitochondrial dysfunction significantly abrogated the effect of oligomycin on GLUT4 protein expression in 3T3-L1 adipocytes (Figure 7E). Therefore, it can be exemplified that the amelioration properties possess by celastrol on the inhibition of NF-κB pathways are correlated with the translocation of GLUT4. Our study is in agreement with another report utilizing streptozotocin-induced diabetic Sprague-Dawley rats by specifying that the inhibition of NF-κB activity elevates the expression of GLUT4 in uterus of diabetic rats [62], indicating the roles of NF-κB may be allied with the relative expression of GLUT-4.

GLUT1 is another type of membrane protein in glucose transporter family that ubiquitously express for indicating basal glucose uptake. The stimulation of AMP-activated protein kinase (AMPK) is reportedly coupled to the enhancement of GLUT1-mediated glucose transport [63]. Furthermore, activation of ERK also leads to the up-regulated expression of GLUT1, resulting in the elevation of basal glucose uptake in the absence of insulin stimulation. Presently, we found no significant changes on the GLUT1 protein level neither in the oligomycin treated cell nor with co-treatment of celastrol. Our data is consistent with the study done by Wang *et al.* [31]. We believe that the mechanism behind this observation is due to intrinsic function of GLUT1 to compensate the maintenance of relative basal glucose uptake in the cell. It was reported that an enhancement of GLUT1 protein level was observed in human cells with mitochondrial dysfunction [64]. Our result indicated that the expression of this protein level was unchanged, specifying that GLUT1 was not involved in NF-κB pathways of adipocytes with mitochondrial dysfunction (Figure 7E). 

The primary goal of our work is to investigate the *in-vitro* mechanism of NF-κB inhibition in the development of mitochondrial dysfunction induced insulin resistance in differentiated 3T3-L1 adipocytes. The limitation of the present study is the use of in-vitro adipocytes to study the effects of NF-κB pathway inhibition in adipocytes with mitochondrial dysfunction. It is clear from our own understanding that further *in vivo* study utilizing genetically modified cells (knockout, over or down-regulation of the NF-κB gene) will better characterize the roles of NF-κB pathways in the development of mitochondrial dysfunction-induced insulin resistance. However, we believe that our works have much value of expositing the specific mechanistic interaction of this regulatory pathway in the development of such perturbations. We hope that this study will be an important reference for therapeutic intervention of diabetes, especially for those diabetes patients with inflammatory disease. Further experimental methodologies in systems-based approaches should be continued to explore the underlying mechanisms of such impairments. The systemic interaction involving whole body homeostasis may represent the holistic assessment of disease mechanisms from the metabolic understanding point of views [65].

## 3. Experimental Section

### 3.1. Cell Culture

3T3-L1 pre-adipocytes were purchased from the American Type Culture Collection (ATCC) (Manassas, VA, USA) and grown in Dulbecco’s Modified Eagle Medium (DMEM) with high glucose (Gibco, Carlsbad, CA, USA) supplemented with 10% of fetal calf serum (FCS) and 1% of penicillin, streptomycin (antibiotics) (Gibco). The cells were sub-cultured every three days before the culture becomes fully confluent, which are about 70%–80% of sub-confluent culture. The standard seeding density used was 2 × 10^5^–4 × 10^5^ cells/cm^2^ and seeded in a 75 cm^2^ flask followed by incubated at 37 °C in the humidified atmosphere of 5% CO_2_. The 3T3-L1 pre-adipocytes were differentiated into adipocytes using a standard protocol [66]. Briefly, the cells were incubated for 48 h before undergo full differentiation. Later, cells were maintained in regular growth medium supplemented with adipogenic cocktail containing MDI (0.5 mM 3-isobutyl-1-methyl-xanthine (IBMX), 0.25 μM dexamethasone and 1 μg/mL insulin) (Sigma, St. Louis, MO, USA) for 2 days. On the 4th day, the media was replaced for every two days with DMEM containing 10% fetal bovine serum (FBS) till 8th day of adipocytes differentiation. In differentiated adipocytes, certain experiments involved stimulation with insulin (10 μg/mL) for 30 min at 37 °C. To avoid confounding variables, the protocol used for insulin stimulation was similar to all types of experiments involving insulin-signaling activities in order. The insulin stimulation is necessary to 3T3-L1 adipocytes in order to measure the glucose uptake and its metabolic activities relative basal level. As insulin resistance is associated with reduced glucose uptake and deteriorated insulin-signaling activities, the experimental approach with insulin stimulation is crucial to observe the underlying effects of such inhibitors on 3T3-L1 adipocytes. According to the standard protocol used widely for treating 3T3-L1 adipocytes, pharmacological level of insulin (10 μg/mL) induce mitosis in adipocytes and 30 min treatment prior to any assays would be useful to observe the effects on insulin-signaling activities [67]. Therefore, insulin dose of 10 µg/mL was chosen for all experiments involving insulin stimulation effects.

### 3.2. Oligomycin and Celastrol Treatment

The establishment of mitochondrial dysfunction-mediated insulin resistance in 3T3-L1 adipocytes was performed as previously described [30]. On the 8th day after induction, fully differentiated 3T3-L1 adipocytes were serum starved for 18 h. The goal of serum starvation is to synchronize the cell cycle. The cells were serum starved before the treatment was performed. Then, the cell-based assays were performed accordingly. Oligomycin (Sigma), ATP synthase inhibitor was used to induce mitochondrial dysfunction in adipocytes. Cells were treated with oligomycin with or without celastrol (Sigma). The cells without treatment were used as controls. Media in control and treated cells were replaced daily, and assays were executed after 9th or 10th day of post-differentiations.

### 3.3. Measurement of Cell Viability

Cell viability was assessed by MTT assay. 3T3-L1 adipocytes were grown in 96-well plates. Cells were then incubated with varying concentration of oligomycin (5, 10, 20 and 30 µM) with or without celastrol (5, 10, 20 and 30 μM) in DMSO. Our preliminary study revealed that the use of DMSO in growth media did not affect the cell viability of adipocytes differentiation at a final concentration of 0.1% (data not shown). To ensure the integrity of adipocytes functions, the optimal dose for each inhibitor that did not significantly affect cell viability was chosen for time-course evaluation. The time-course dependent was evaluated for each optimal dose (12, 24, 36 and 48 h). Briefly, phosphate buffer saline (PBS) was used to dissolve 5 mg/mL MTT before filtered through 0.2 μM micro-filter and will be stored at 4 °C. Fully differentiated 3T3-L1 adipocytes were washed out with PBS. 10 μL of MTT stock solution was prepared and subsequently added into each well and incubated for 3–4 h at 37 °C. After that, 200 μL of dimethyl sulfoxide (DMSO) was transferred into each well to dissolve the insoluble purple formazan product into coloured solution. The absorbance of the colored solution was measured at the wavelength of 570 nm and reference wavelength of 630 nm using the ELISA plate reader (Erba LisaScan II, Mannheim, Germany). Optical densities of treated cells were normalized to controls. Each condition was performed in triplicate.

### 3.4. Intracellular ATP Concentration

Intracellular ATP concentrations were assayed by using a calorimetric ATP Assay Kit (KA0806; Abnova Corporation, Walnut, CA, USA). The cells were lysed in 100 μL ATP Assay Buffer followed by deproteinization of cell lysates using 10 kDa Spin Column. Using ATP standard, intracellular ATP concentrations were then determined with ELISA plate reader measured at the wavelength of 570 nm and normalized to protein concentrations.

### 3.5. Measurement of Mitochondrial Membrane Potentials (ΔΨm)

The measurement of mitochondrial membrane potential were determined using the mitochondria-specific lipophilic cationic fluorescence dye 5,5',6,6'-tetrachloro-1,1',3,3'-tetraethylbenzimi-dazolylcarbocyanine iodide (JC-1) Detection Kit (KA1324; Abnova Corporation) following the manufacturer’s instruction. Briefly, treated 1 × 10^6^ cells were plated on 96-well black culture plate in 100 μL culture medium in a CO_2_ incubator overnight at 37 °C. After then, 10 μL of the JC-1 Staining Solution were added to each well and incubated in CO_2_ incubator at 37 °C for 15–30 min. The plate was centrifuged for 5 min at 400× *g* at room temperature and supernatant was discarded. 100 μL of Assay Buffer was added to each well and ready for analysis by a fluorescent plate reader (Promega, Madison, WI, USA). J-aggregates were formed in healthy cells with excitation and emission at 560 and 595 nm, respectively. In unhealthy cells, JC-1 exists as monomers that was excited at 485 nm and emits at 535 nm.

### 3.6. Mitochondrial Superoxide Measurement

Cells in black-walled 96-well plates were treated with oligomycin and celastrol for 48 h as described above. ROS production in adipocytes was measured by incubating cells with 1 µM Mitosox Red (Invitrogen Molecular Probes, Carlsbad, CA, USA) for 30 min at 37 °C. After two times washes with PBS, cells were collected and used for fluorescence analysis using a multimode plate reader at an excitation wavelength of 510 nm and an emission wavelength of 580 nm.

### 3.7. Immunoblotting

Western blotting analysis was carried out as previously described [68]. Briefly, after treatments, the cells were washed twice with Krebs-Ringer HEPES buffer and homogenized with protein lysis buffer (50 mM Tris pH 7.5, 1 mM EDTA, 1 mM EGTA, 10% glycerol, 1% triton X-100, 50 mM NaF, 5 mM Na_4_P_2_O_7_, 1 mM Na_3_VO_4_, 1 mM DTT) containing protease and phosphatase inhibitors. The cell lysate was incubated on ice for about 1 h with constant mixing. Proteins concentrations were quantified using a Pierce™ BCA Protein Assay Kit (Thermo Scientific, Waltham, MA, USA). To be used as a whole protein, supernatants were collected after centrifugation at 16,000× *g* for 20 min at 4 °C. The aliquots of whole protein was separated on odecyl sulfate-polyacrylamide gel electrophoresis (SDS-PAGE), and transferred to nitrocellulose membranes (Whatman, London, UK). Membrane were blocked with Tris-buffered saline/Tween-20 (TBST, 0.14 mol/L NaCl, 0.02 mol/L Tris base (pH 7.6), and 0.1% Tween) containing 5% *w*/*v* bovine serum albumin (BSA) for 1 h at room temperature. After then, the membranes were incubated overnight at 4 °C with specific primary antibodies of interest, at the dilution indicated, in TBST: NF-κB p65 (1:1000, Cell Signaling Technology, Danvers, MA, USA), mfn1 (1:1000, Santa Cruz Biotechnology, Santa Cruz, CA, USA), mfn2 (1:1000, Santa Cruz Biotechnology), drp1 (1:1000, Santa Cruz Biotechnology), pY612-IRS1 (1:1000, Upstate Biotechnology, Lake Placid, New York, NY, USA), IRS1 (1:1000, Upstate), pS473-Akt (1:1000, Cell Signaling Technology), Akt (1:1000, Cell Signaling Technology), pS462 AS160 (1:1000, Cell Signaling Technology), AS160 (1:1000, Cell Signaling Technology), GLUT4 (1:1000, Cell Signaling Technology), GLUT1 (1:1000, Novus Biologicals, Littleton, CO, USA) and β-actin (1:1000, Cell Signaling Technology) at room temperature. Membranes were washed with TBST at a dilution of 1: 10,000 for 1 h at room temperature before the corresponding horseradish peroxidase (HRP)-conjugated secondary antibodies (Pierce Biotechnology, Rockford, IL, USA) were treated. Specific immune complexes were detected by chemiluminescence using ECL plus kits (Amersham, Piscataway, NJ, USA), visualized using ChemiDoc™ XRS+ (Bio-Rad, Hercules, CA, USA) with Image Lab™ software (Bio-Rad, Hercules, CA, USA) and quantified by using a standard densitometry.

### 3.8. Measurement of DNA Oxidative Damage and Lipid Peroxidation

8-OHdG is a ubiquitous marker of oxidative stress-mediated DNA damage, was quantified using the OxiSelect™ Oxidative DNA Damage ELISA Kit (Cell Biolabs, San Diego, CA, USA) as previously described [69]. The lipid peroxidation was assessed by measuring malondialdehyde (MDA) as an end product of lipid peroxidation using Lipid Peroxidation (MDA) Assay Kit (Sigma) according to the manufacturer’s protocols.

### 3.9. Protein Carbonylation

Protein carbonyls were detected using Protein Carbonyl Content Assay Kit (Cayman, Ann Arbor, MI, USA). Briefly, cells were washed with PBS and centrifuged to 1000–2000× *g* for 10 min at 4 °C. The cell pellet was sonicated on ice in 2 mL of cold buffer containing 50 mM phosphate, pH 6.7 with 1 mM EDTA. Then, the mixture was centrifuged again at 10,000× *g* for 15 min at 4 °C and supernatant was removed and stored on ice. After that, 800 µL of DPNH and 2.5 M HCI was added to sample and control tubes, respectively. The tubes were incubated for 1 h at dark room temperature followed by the addition of 1 mL of 20% TCA to each tube on ice. All tubes were centrifuged at 10,000× *g* for 10 min at 4 °C and supernatant was discarded. The cell pellet was re-suspended in 1 mL of (1:1) ethanol/ethyl acetate mixture. These steps were repeated three times in order to remove cellular debris. After the final wash, the protein pellet was re-suspended in 500 µL of guanidine hydrochloride and centrifuged at the same speed as stated above to remove any leftover debris. Each 220 µL of sample and control tubes were transferred to 96-well plates and measured at the absorbance of 385 nm using a plate reader.

### 3.10. Measurement of Lipolysis

Lipolysis was evaluated by measuring the amount of glycerol and free fatty acids released into the media. In brief, lipolysis was evaluated by measuring the amount of glycerol and free fatty acids released to the media. Glycerol level was determined after 48 h of oligomycin and celastrol treatments while free fatty acids release were quantified after 3 h of drugs treatment by using the Lipolysis Assay Kit for Free Fatty Acids and Glycerol Detection (Zen-Bio Inc, Research Triangle Park, NC, USA) according to the manufacturer’s instructions.

### 3.11. Quantification of Lipid Contents by Oil Red O Assay

Intracellular accumulations of lipid droplets were determined by oil red O staining. This assay was performed as previously mentioned [66]. Briefly, 0.7 g of oil red O was dissolved in 200 mL of isopropanol and filtered with 0.2 µm. The solution was diluted into a ratio of 6:4 with distilled water. The solution was then mixed and allowed to stand for 20 min at room temperature. The cells were washed with 10% formalin in PBS and stained with a filtered staining solution for 10 min at 37 °C. The stained cells were washed again three times with distilled water and observed under inverted fluorescence microscope (Carl Zeiss, Göttingen, Germany) equipped with the digital camera AxioCam MRc (Carl Zeiss) for morphological study. For quantification of lipid content, the oil red O was eluted by adding 100% isopropanol and incubated for 10 min before measuring the absorbance (OD) at 490 nm using ELISA plate reader.

### 3.12. Cytokine ELISA for TNF-α and IL-1β Measurement

The concentrations of pro-inflammatory cytokines TNF-α and IL-1β in the harvested medium from adipocytes were measured using a commercially available enzyme-linked immunosorbent assay (ELISA) kit (Invitrogen, Carlsbad, CA, USA) according to the manufacturer’s protocol. The minimum detectable doses were 7 pg/mL for IL-1β and 3 pg/mL for TNF-α. Each concentration was obtained from the standard curve and expressed as per mg of total extractable cell protein.

### 3.13. Glucose Uptake Assay

To evaluate increased dependence on glycolytic metabolism in the presence of mitochondrial inhibitors, glucose uptake activity was performed by quantifying the uptake of radio-labelled glucose. Briefly, the differentiated cells with and without treatment were grown in 12-well plates and washed twice with serum-free media and incubated for 3 h at 37 °C. The cells were washed three times with Krebs-Ringer HEPES (KRPH) buffer and undergo incubation with 0.9 mL of KRPH buffer for 30 min at 37 °C. The activity of glucose uptake was quantified by the addition of 0.1 mL of KRPH buffer comprising 2-deoxy-d-[^3^H] glucose (0.037 MBq; Perkin Elmer, Foster City, CA, USA) and glucose (0.001 mM). The glucose uptake activity was terminated right after 60 min, through washing the cells three times with ice-cold PBS. The cells were lysed through incubation for 20 min at 37 °C with 0.7 mL of 1% Triton X-100. The radioactivity level in the cell lysates were quantified by using Tri-Carb 2700TR liquid scintillation counter.

### 3.14. Statistical Analysis

Experimental results were shown as the mean means ± standard error of the mean (SEM) of the indicated sample size (*n*). Statistical significance of data was determined using paired and unpaired Student’s *t*-test. One-way ANOVA with Tukey post-hoc was utilized where more than two groups were compared with treatments. A value of *p* < 0.05 was considered as statistically significant.

## 4. Conclusions

In summary, based on the results obtained in this study, we have proposed additional support to the hypothesis that a decline in mitochondrial function is significantly associated with differentiated 3T3-L1 adipocytes inflammatory phenotype observed in insulin resistance-associated diseases. The results of the present study verify that mitochondrial dysfunction alone may disturb oxidative metabolism and promotes low-grade inflammation in adipocytes. These metabolic insults were ameliorated by the addition of celastrol on the differentiated adipocytes with mitochondrial dysfunction. To our best knowledge, this is the first report demonstrating deterioration of mitochondrial function in adipocytes with insulin resistance is correlated with the activation of NF-κB pathways. The limitation of the present study is the use of *in vitro* model of 3T3-L1 adipocytes to observe such effects on the ameliorative properties of NF-κB inhibitor. The use of different methodologies in further evaluating NF-κB expression activity is imperatively needed. In addition, future analyses involving *in vivo* and human studies should be continued to explicate the role of this inflammatory signaling pathway in these metabolic perturbations. However, our results have substantiated the mechanistic role of inflammation in the dysregulation of oxidative metabolism in adipose tissues. The study have provided valuable information for the development of an envisaged therapeutic strategy in insulin resistance and type 2 diabetes.
